# Supplementary material for: Modeling glioblastoma heterogeneity as a dynamic network of cell states
Source: Mol Syst Biol. 2021 Sep 16;17(9):e10105. doi: 10.15252/msb.202010105 (PMC8444284; doi:10.15252/msb.202010105)
Supplement: Supplementary file 6 — Source Data for Figure 5 [file MSB-17-e10105-s004.zip › Figure5A_sourcedata/GSEA_3071/nosigdb_stateIV.GseaPreranked.1622795164626/RICHARDS_NOWAKOWSKI_SCIENCE_2017_NIN1_UPREG.html]

Details for gene set RICHARDS\_NOWAKOWSKI\_SCIENCE\_2017\_NIN1\_UPREG[GSEA]

|  || Dataset | state53071 |
| Phenotype | NoPhenotypeAvailable |
| Upregulated in class | na\_pos |
| GeneSet | RICHARDS\_NOWAKOWSKI\_SCIENCE\_2017\_NIN1\_UPREG |
| Enrichment Score (ES) | 0.58150774 |
| Normalized Enrichment Score (NES) | 3.2557929 |
| Nominal p-value | 0.0 |
| FDR q-value | 0.0 |
| FWER p-Value | 0.0 |
Table: GSEA Results Summary

  

Fig 1: Enrichment plot: RICHARDS\_NOWAKOWSKI\_SCIENCE\_2017\_NIN1\_UPREG      
 Profile of the Running ES Score & Positions of GeneSet Members on the Rank Ordered List

  

| PROBE | GENE SYMBOL | GENE\_TITLE | RANK IN GENE LIST | RANK METRIC SCORE | RUNNING ES | CORE ENRICHMENT || 1 | DCX |  |  | 4 | 2.752 | 0.0289 | Yes |
| 2 | DLX5 |  |  | 17 | 2.283 | 0.0513 | Yes |
| 3 | PLXNA4 |  |  | 20 | 2.236 | 0.0750 | Yes |
| 4 | DLX2 |  |  | 50 | 1.858 | 0.0895 | Yes |
| 5 | SOX11 |  |  | 56 | 1.811 | 0.1081 | Yes |
| 6 | DLX1 |  |  | 62 | 1.768 | 0.1262 | Yes |
| 7 | CDH2 |  |  | 68 | 1.735 | 0.1439 | Yes |
| 8 | DCLK2 |  |  | 74 | 1.704 | 0.1614 | Yes |
| 9 | DPYSL3 |  |  | 75 | 1.691 | 0.1796 | Yes |
| 10 | ZBTB20 |  |  | 81 | 1.628 | 0.1962 | Yes |
| 11 | TCF4 |  |  | 96 | 1.551 | 0.2103 | Yes |
| 12 | NEDD4L |  |  | 100 | 1.535 | 0.2263 | Yes |
| 13 | RAB3IP |  |  | 124 | 1.474 | 0.2377 | Yes |
| 14 | NCAM1 |  |  | 129 | 1.456 | 0.2527 | Yes |
| 15 | LSAMP |  |  | 130 | 1.454 | 0.2684 | Yes |
| 16 | CCDC88A |  |  | 135 | 1.429 | 0.2830 | Yes |
| 17 | CHD7 |  |  | 144 | 1.386 | 0.2965 | Yes |
| 18 | DLX6 |  |  | 145 | 1.384 | 0.3114 | Yes |
| 19 | BCL11A |  |  | 154 | 1.367 | 0.3246 | Yes |
| 20 | BCL11B |  |  | 163 | 1.343 | 0.3376 | Yes |
| 21 | ZEB2 |  |  | 164 | 1.339 | 0.3520 | Yes |
| 22 | TUBB3 |  |  | 167 | 1.330 | 0.3660 | Yes |
| 23 | NRXN3 |  |  | 170 | 1.321 | 0.3799 | Yes |
| 24 | CDK6 |  |  | 178 | 1.299 | 0.3925 | Yes |
| 25 | SP9 |  |  | 189 | 1.281 | 0.4044 | Yes |
| 26 | GAD1 |  |  | 190 | 1.281 | 0.4183 | Yes |
| 27 | SLAIN1 |  |  | 203 | 1.257 | 0.4295 | Yes |
| 28 | SOX6 |  |  | 207 | 1.244 | 0.4424 | Yes |
| 29 | GOLM1 |  |  | 209 | 1.240 | 0.4556 | Yes |
| 30 | SOX2 |  |  | 212 | 1.231 | 0.4685 | Yes |
| 31 | CADPS |  |  | 220 | 1.205 | 0.4801 | Yes |
| 32 | RBP1 |  |  | 254 | 1.136 | 0.4860 | Yes |
| 33 | MEIS1 |  |  | 269 | 1.107 | 0.4952 | Yes |
| 34 | PFN2 |  |  | 270 | 1.107 | 0.5072 | Yes |
| 35 | ARL4D |  |  | 295 | 1.039 | 0.5138 | Yes |
| 36 | PDZRN4 |  |  | 318 | 1.015 | 0.5205 | Yes |
| 37 | SOX4 |  |  | 377 | 0.928 | 0.5193 | Yes |
| 38 | PDZRN3 |  |  | 405 | 0.904 | 0.5238 | Yes |
| 39 | KLHL13 |  |  | 412 | 0.893 | 0.5323 | Yes |
| 40 | ARX |  |  | 415 | 0.891 | 0.5416 | Yes |
| 41 | BEST3 |  |  | 457 | 0.849 | 0.5428 | Yes |
| 42 | ANTXR1 |  |  | 462 | 0.847 | 0.5512 | Yes |
| 43 | ZNF704 |  |  | 498 | 0.818 | 0.5532 | Yes |
| 44 | HDAC9 |  |  | 510 | 0.810 | 0.5598 | Yes |
| 45 | B4GALT5 |  |  | 541 | 0.774 | 0.5624 | Yes |
| 46 | ST8SIA4 |  |  | 567 | 0.751 | 0.5657 | Yes |
| 47 | FNBP1L |  |  | 576 | 0.741 | 0.5721 | Yes |
| 48 | ELAVL3 |  |  | 582 | 0.739 | 0.5792 | Yes |
| 49 | GLCCI1 |  |  | 611 | 0.719 | 0.5815 | Yes |
| 50 | HIGD1A |  |  | 720 | 0.648 | 0.5676 | No |
| 51 | MALAT1 |  |  | 739 | 0.638 | 0.5710 | No |
| 52 | CDCA7 |  |  | 818 | 0.596 | 0.5624 | No |
| 53 | COL4A3BP |  |  | 825 | 0.590 | 0.5676 | No |
| 54 | HIPK2 |  |  | 896 | 0.563 | 0.5602 | No |
| 55 | ARID4B |  |  | 903 | 0.559 | 0.5650 | No |
| 56 | PCDH17 |  |  | 956 | 0.541 | 0.5608 | No |
| 57 | SENP7 |  |  | 989 | 0.526 | 0.5603 | No |
| 58 | SRGAP2B |  |  | 1005 | 0.520 | 0.5630 | No |
| 59 | KIAA1958 |  |  | 1029 | 0.513 | 0.5641 | No |
| 60 | QKI |  |  | 1078 | 0.500 | 0.5602 | No |
| 61 | BTG1 |  |  | 1123 | 0.486 | 0.5570 | No |
| 62 | GNPTAB |  |  | 1127 | 0.486 | 0.5617 | No |
| 63 | AP2M1 |  |  | 1164 | 0.476 | 0.5598 | No |
| 64 | NBEA |  |  | 1239 | 0.454 | 0.5504 | No |
| 65 | DCAF7 |  |  | 1251 | 0.452 | 0.5532 | No |
| 66 | SMARCD1 |  |  | 1322 | 0.435 | 0.5444 | No |
| 67 | PHF20L1 |  |  | 1360 | 0.426 | 0.5418 | No |
| 68 | CSNK1E |  |  | 1412 | 0.413 | 0.5364 | No |
| 69 | MAPK10 |  |  | 1471 | 0.398 | 0.5295 | No |
| 70 | FXR1 |  |  | 1531 | 0.382 | 0.5222 | No |
| 71 | GRIA4 |  |  | 1545 | 0.379 | 0.5238 | No |
| 72 | MOB3B |  |  | 1552 | 0.377 | 0.5267 | No |
| 73 | GLUL |  |  | 1582 | 0.370 | 0.5251 | No |
| 74 | ZFHX2 |  |  | 1614 | 0.366 | 0.5231 | No |
| 75 | DMTF1 |  |  | 1637 | 0.361 | 0.5227 | No |
| 76 | MTRNR2L1 |  |  | 1697 | 0.350 | 0.5151 | No |
| 77 | MARCKS |  |  | 1713 | 0.348 | 0.5159 | No |
| 78 | CNOT7 |  |  | 1714 | 0.347 | 0.5197 | No |
| 79 | ERBB4 |  |  | 1843 | 0.325 | 0.4984 | No |
| 80 | PHF21B |  |  | 1855 | 0.323 | 0.4998 | No |
| 81 | RC3H2 |  |  | 1872 | 0.318 | 0.5001 | No |
| 82 | MFN1 |  |  | 1891 | 0.315 | 0.5001 | No |
| 83 | CREB1 |  |  | 1936 | 0.307 | 0.4949 | No |
| 84 | ELAVL2 |  |  | 1938 | 0.307 | 0.4980 | No |
| 85 | EPHA4 |  |  | 2032 | 0.292 | 0.4832 | No |
| 86 | TOX3 |  |  | 2039 | 0.290 | 0.4851 | No |
| 87 | TET2 |  |  | 2082 | 0.286 | 0.4801 | No |
| 88 | PDE4D |  |  | 2100 | 0.283 | 0.4799 | No |
| 89 | STAT2 |  |  | 2113 | 0.282 | 0.4806 | No |
| 90 | ATP6V1G1 |  |  | 2148 | 0.277 | 0.4770 | No |
| 91 | VEZF1 |  |  | 2161 | 0.274 | 0.4777 | No |
| 92 | TSPYL4 |  |  | 2189 | 0.269 | 0.4753 | No |
| 93 | MAF |  |  | 2206 | 0.267 | 0.4751 | No |
| 94 | MIR181A1HG |  |  | 2218 | 0.266 | 0.4759 | No |
| 95 | PPHLN1 |  |  | 2229 | 0.264 | 0.4768 | No |
| 96 | TOX |  |  | 2285 | 0.258 | 0.4690 | No |
| 97 | ZNF37A |  |  | 2301 | 0.255 | 0.4688 | No |
| 98 | GART |  |  | 2337 | -0.251 | 0.4648 | No |
| 99 | PSMA2 |  |  | 2490 | -0.268 | 0.4383 | No |
| 100 | EIF1AX |  |  | 2584 | -0.280 | 0.4233 | No |
| 101 | DOCK11 |  |  | 2627 | -0.286 | 0.4183 | No |
| 102 | ZC3H8 |  |  | 2870 | -0.315 | 0.3749 | No |
| 103 | ZNF618 |  |  | 2922 | -0.321 | 0.3685 | No |
| 104 | ABCA1 |  |  | 3078 | -0.342 | 0.3423 | No |
| 105 | MYO1B |  |  | 3431 | -0.396 | 0.2785 | No |
| 106 | PSMG2 |  |  | 3459 | -0.401 | 0.2776 | No |
| 107 | HIPK1 |  |  | 3553 | -0.419 | 0.2642 | No |
| 108 | TRAM1 |  |  | 3699 | -0.445 | 0.2409 | No |
| 109 | DIMT1 |  |  | 3915 | -0.490 | 0.2047 | No |
| 110 | EHBP1 |  |  | 3972 | -0.504 | 0.1993 | No |
| 111 | ADAMTS5 |  |  | 4047 | -0.525 | 0.1907 | No |
| 112 | RND3 |  |  | 4402 | -0.635 | 0.1291 | No |
| 113 | ALYREF |  |  | 4406 | -0.638 | 0.1354 | No |
| 114 | CCNE2 |  |  | 4564 | -0.714 | 0.1128 | No |
| 115 | MIAT |  |  | 4622 | -0.748 | 0.1098 | No |
| 116 | PDE4DIP |  |  | 4715 | -0.804 | 0.1007 | No |
| 117 | ACTG1 |  |  | 4882 | -0.947 | 0.0789 | No |
Table: GSEA details [plain text format]

  

Fig 2: RICHARDS\_NOWAKOWSKI\_SCIENCE\_2017\_NIN1\_UPREG: Random ES distribution      
 Gene set null distribution of ES for **RICHARDS\_NOWAKOWSKI\_SCIENCE\_2017\_NIN1\_UPREG**
